# On-Chip Sensing of Thermoelectric Thin Film’s Merit

**DOI:** 10.3390/s150717232

**Published:** 2015-07-16

**Authors:** Zhigang Xiao, Xiaoshan Zhu

**Affiliations:** 1 Department of Electrical Engineering, Alabama A&M University, Normal, AL 35762, USA; 2 Department of Electrical and Biomedical Engineering, University of Nevada, Reno, NV 89557, USA; E-Mail: xzhu@unr.edu

**Keywords:** silicon diode temperature sensors, thermoelectric thin films and devices, Sb_2_Te_3_ thin film, microfabrication

## Abstract

Thermoelectric thin films have been widely explored for thermal-to-electrical energy conversion or solid-state cooling, because they can remove heat from integrated circuit (IC) chips or micro-electromechanical systems (MEMS) devices without involving any moving mechanical parts. In this paper, we report using silicon diode-based temperature sensors and specific thermoelectric devices to characterize the merit of thermoelectric thin films. The silicon diode temperature sensors and thermoelectric devices were fabricated using microfabrication techniques. Specifically, e-beam evaporation was used to grow the thermoelectric thin film of Sb_2_Te_3_ (100 nm thick). The Seebeck coefficient and the merit of the Sb_2_Te_3_ thin film were measured or determined. The fabrication of silicon diode temperature sensors and thermoelectric devices are compatible with the integrated circuit fabrication.

## 1. Introduction

**Figure 1:**
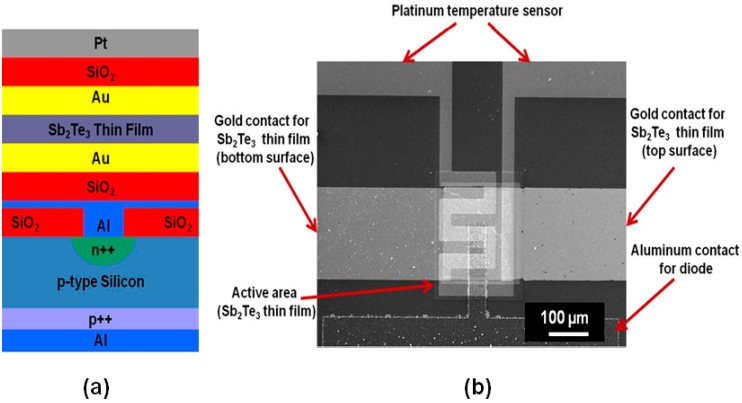
(**a**) Schematic of the cross-section of a cross-plane TE device for measurement of the cross-plane Seebeck coefficient of Sb_2_Te_3_ thin film; (**b**) SEM image of the fabricated TE device.

**Figure 2:**
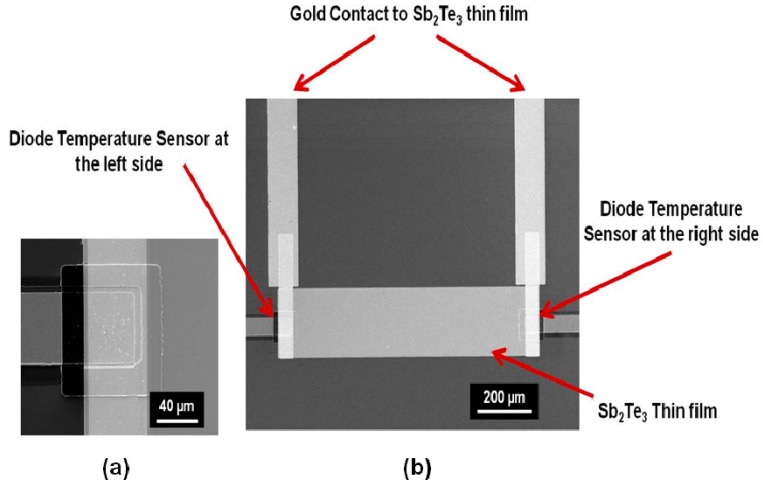
(**a**) SEM image of an enlarged view on a diode temperature sensor; (**b**) SEM image of a fabricated TE device for measurement of the in-plane Seebeck coefficient of Sb_2_Te_3_ thin film, where two diode temperature sensors were fabricated under the two ends of the Sb_2_Te_3_ film and separated by an insulation layer of SiO_2_.

**Figure 3:**
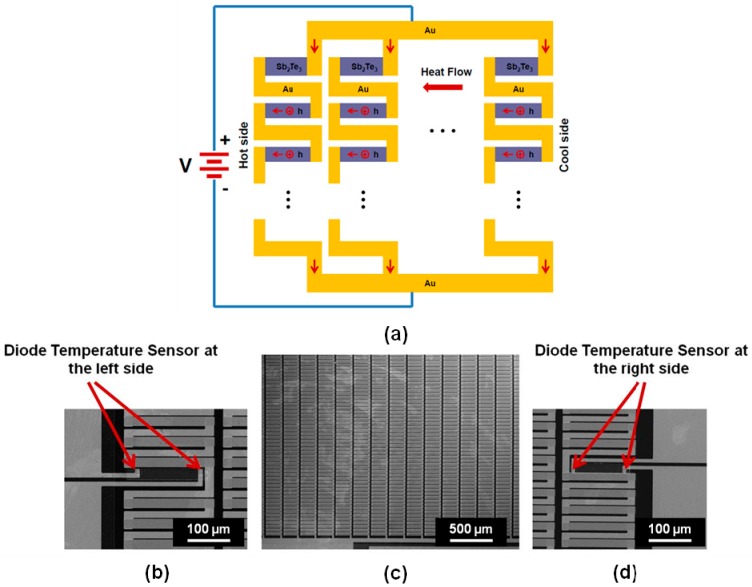
(**a**) Working principle for an in-plane integrated TE device of Sb_2_Te_3_ thin film; (**b**) SEM image of an enlarge view on the area of left diode temperature sensors in the integrated TE device; (**c**) SEM image of a partial of the fabricated integrated TE device; (**d**) SEM image of an enlarge view on the area of right diode temperature sensors in the integrated TE device.

Managing heat flux has been one of the most important technical challenges that the current integrated circuit (IC) or MEMS industry is facing, because the rising temperature limits the miniaturization of IC or MEMS devices and decreases their lifetime [1]. Solid-state thermoelectric (TE) cooling techniques are of great interest to solve this problem, because they can remove heat from the IC chips or MEMS devices without involving any moving mechanical parts. TE materials and devices have been extensively investigated by researchers for the application of thermal-to-electrical energy conversion or solid-state cooling [2,3,4,5,6,7,8,9,10,11,12,13,14,15,16]. However, the use of TE materials and devices is currently limited by their low efficiency. Nanostructured TE materials, such as nanolayered thin films and quantum-dot superlattices, have been found to have a thermoelectric merit significantly greater than the same bulk materials [17], due to the effect of quantum confinement on electrons and holes, as well as higher impedance to phonon transport in the nanoscale materials. The thermoelectric merit is defined as: *ZT = S^2^*σ*T/*κ, where *T* is the absolute temperature, σ is the electrical conductivity, κ is the thermal conductivity, and *S* is the Seebeck coefficient (defined as voltage difference divided by temperature difference (Δ*V/*Δ*T*)). The figure of merit of a TE device can also be obtained by measuring the maximum temperature achieved in the device. The formula for calculating the *ZT* value of a TE device is: *ZT =* 2Δ*T_max_/T*. It is challenging to characterize the nanoscale TE materials and accurately measure the three parameters simultaneously for finding the *ZT* value. On the other hand, in some TE material based devices, it is desired to frequently in-field monitor the *ZT* change of the TE materials in order to diagnose the device lifetime or quality degradation in use. To address these needs, two microstructures and one in-plane integrated TE device integrating temperature sensors and Sb_2_Te_3_ film, are proposed for on-chip sensing of the Seebeck Coefficient and *ZT* parameter (as shown in Figure 1, Figure 2 and Figure 3). To measure the cross-plane Seebeck coefficient of a TE film, the TE film is sandwiched between a diode-based temperature sensor and a Pt film temperature sensor (Figure 1). The cross-plane Seebeck coefficient is obtained by measuring the voltage difference and temperature difference between the top and bottom surface of the film. To measure the in-plane Seebeck coefficient of a TE film, the two ends of the TE film are located on the top of two diode-based temperature sensors (Figure 2). The in-plane Seebeck coefficient is obtained by measuring the voltage difference and temperature difference between the two ends of the film. To measure the *ZT* value of the in-plane integrated TE device, two diode-based temperature sensors are designed at the left side and the right side of the device (Figure 3). The *ZT* value is obtained by measuring the maximum temperature difference achieved in the TE device. These microstructures are fully compatible to IC or MEMS fabrication steps, and can be easily built in to any TE film based devices.

## 2. Experimental Details

We fabricated three thermoelectric (TE) devices of Sb_2_Te_3_ thin film to characterize the silicon diode temperature sensors and measure the in-plane and cross-plane Seebeck coefficient of Sb_2_Te_3_ thin film using the microfabrication techniques. The three TE devices include two single TE devices, and one integrated TE device, which consists of many TE elements. A 76-mm-diameter p-type (001) silicon wafer was used as the substrate in fabricating the TE devices. The silicon wafer has a resistivity of 1 to 5 Ω·cm. The fabrication processes included thermal wet oxidation to growing the initial oxide layer; heavily boron doping on the backside of wafer for making the ohmic contact; phosphorous doping for making the p-n junction of diode temperature sensor; depositing the SiO_2_ thin film for the insulation layer; depositing the Sb_2_Te_3_ thin film for making the TE element; depositing the aluminum (Al) and gold (Au) thin films for the metal contact. Thermal diffusion was used for both boron and phosphorous doping. Ultra-violet (UV) lithography was adopted for patterning in fabricating the devices. All the thin films were grown via electron-beam evaporation at room temperature without substrate heating. The substrate holder was rotated at the speed of 20 RPM during the deposition. The process chamber had a background pressure of 2 × 10^−7^ Torr. An INFICON deposition monitor controlled their thicknesses. Solid antimony (III) telluride (99.999% purity; Alfa Aesar Company) was used as the source material for growth of the Sb_2_Te_3_ thin film. The deposition pressure was 1 × 10^−6^ Torr, and the growth rate was 1 Å/s. The film was prepared as 100 nm thick. The E-beam-evaporation-grown antimony telluride film can have the desired stoichiometry of Sb_2_Te_3_ [18]. A 50 nm-thick gold (Au) layer was deposited for the metal contact of the Sb_2_Te_3_ film. A 100-nm-thick platinum (Pt) layer was grown for fabricating the Pt Serpentine-resistor temperature sensor. The fabricated device wafer was annealed at 200 °C for 30 min before the measurements. The devices were imaged in a scanning electron microscope. All the electrical measurements were performed under the vacuum condition of 5 × 10^−4^ Torr.

## 3. Results and Discussion

Figure 1a is the schematic of the cross-section of the cross-plane TE device for measuring the cross-plane Seebeck coefficient of the Sb_2_Te_3_ thin film, which is sandwiched by a diode temperature sensor and a platinum (Pt) temperature sensor; Figure 1b shows the SEM image of the fabricated cross-plane TE device. The silicon diode temperature sensor centered on the bottom, and the platinum (Pt) serpentine resistor temperature sensor centered on the top. The fabricated cross-plane TE device with pads for the outside contacts has a dimension of 0.7 cm × 0.7 cm, where the active area covered by the Sb_2_Te_3_ film is 200 µm × 200 µm. Figure 2 shows the SEM images of the fabricated in-plane TE device and the diode temperature sensor for measuring the in-plane Seebeck coefficient of the Sb_2_Te_3_ thin film. Two silicon diode temperature sensors were fabricated at the two ends of the film. The fabricated in-plane TE device with pads for the outside contacts has a dimension of 1 cm × 0.6 cm, where the active area covered by the Sb_2_Te_3_ film is 1000 µm × 200 µm. Figure 3a is the working principle for an in-plane integrated TE device of Sb_2_Te_3_ thin film; Figure 3b shows the SEM image of an enlarged view on the area of left diode temperature sensors in the integrated TE device; Figure 3c shows the SEM image of a partial of the fabricated integrated TE device; Figure 3d shows the SEM image of an enlarge view on the area of right diode temperature sensors in the integrated TE device. The two silicon diode temperatures were fabricated at the two sides of the device for measuring the temperature difference. The fabricated integrated TE device with pads for the outside contacts has a dimension of 2.5 cm × 2 cm, where the active area covered by the Sb_2_Te_3_ elements is 1.5 cm × 1 cm. Sb_2_Te_3_ is the thermoelectric material in the device and has the major thermoelectric effect for the device. Gold (Au) is not a thermoelectric material and does not have the thermoelectric effect. The gold layer is the contact material in the device, and can contribute to make a higher *ZT* value for the device by decreasing the contact resistance with the Sb_2_Te_3_ layer. The gold layer does not make any contribution to the Seebeck coefficient.

**Figure 4:**
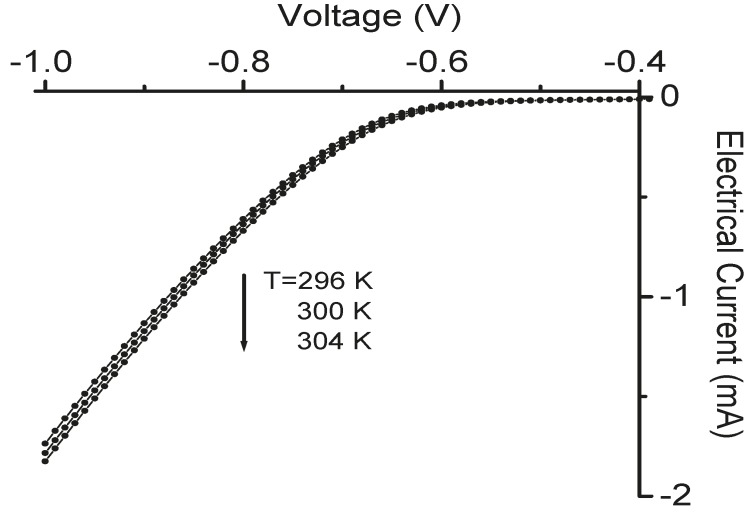
The variation of electrical currents as a function of applied voltages at three temperatures (T = 296 K, 300 K, and 304 K) for a fabricated silicon diode temperature sensor.

**Figure 5:**
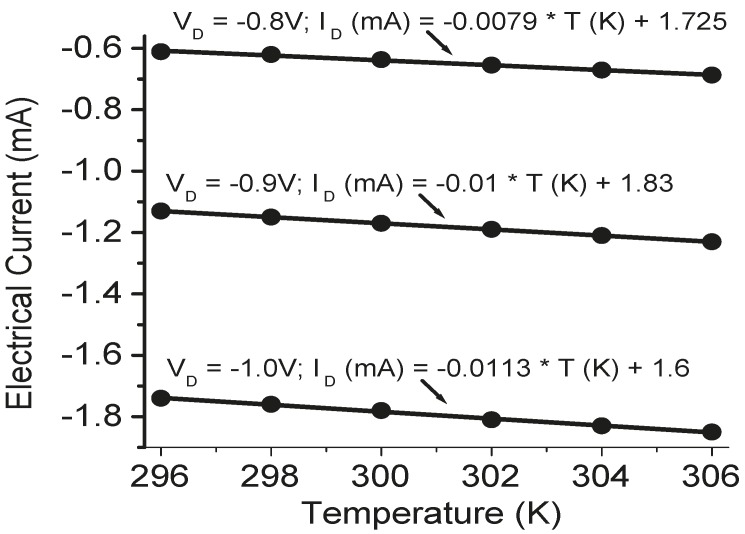
The variation of electrical currents as a function of temperatures at three applied voltages (V_R_ = −0.8 V, −0.9 V, and −1.0 V) for a fabricated silicon diode temperature sensor, where the dots are the experimental data, and the linear current-temperature functions were derived from the regression analysis of the experimental data.

**Figure 6:**
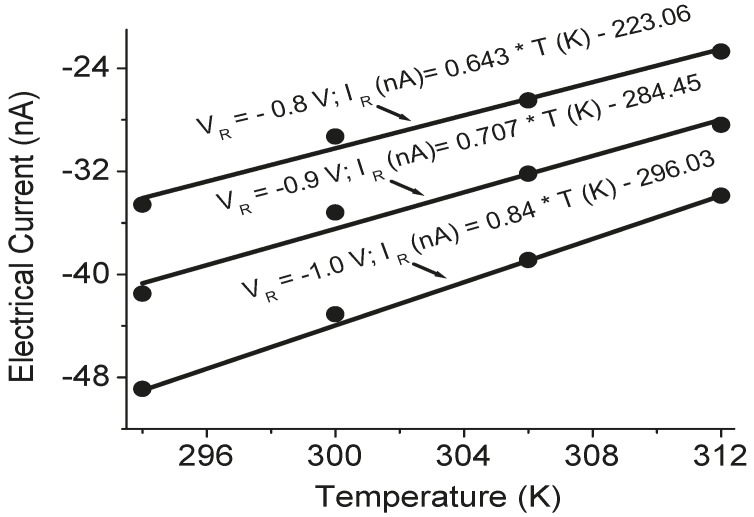
The variation of electrical currents as a function of temperatures at three applied voltage values (V_R_ = −0.8 V, −0.9 V, and −1.0 V) for the platinum resistor temperature sensor in the TE device, where the dots are the experimental data and the linear current-temperature functions were derived from the regression analysis of the experimental data.

**Figure 7:**
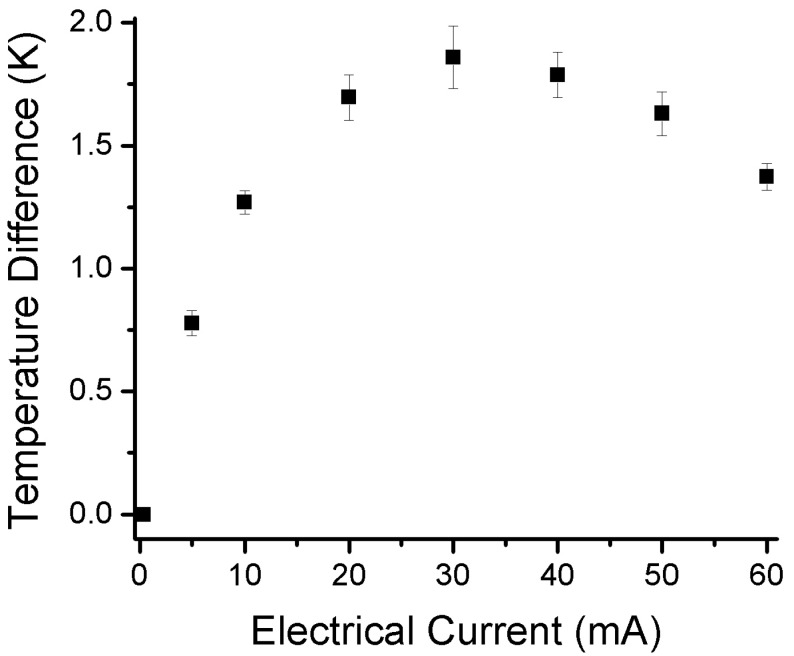
The variation of the temperature difference between the left and right sides of the in-plane Sb_2_Te_3_ integrated thermoelectric device as a function of applied DC electrical currents.

The temperature sensors in the TE devices were calibrated before measurement. The calibration results are shown in Figure 4, Figure 5 and Figure 6. A good linear relation between the electrical current value and temperature was obtained for both Pt serpentine resistor temperature sensor and the silicon diode temperature sensor. The electrical current-voltage (IV) curves in Figure 4 shows the silicon diode temperature sensor has a turn-on voltage of about 0.6 V. The Seebeck coefficient of the Sb_2_Te_3_ thin film was then measured, and the measured cross-plane and in-plane Seebeck coefficients were (98 ± 4.5) µV/K and (105.3 ± 5.1) µV/K, respectively. The temperature difference between the left side and the right side of the integrated TE device was measured from the two silicon diode temperature sensors in the device. Figure 7 shows the temperature difference as a function of applied DC electrical currents. The temperature difference increases to a maximum value of about 1.8 K at an applied DC current of 30 mA with the increase of electrical currents, then decreases with further increase of the currents. When the applied current increases, the heating effect becomes larger and larger. As a result, the temperature difference decreases with further increasing current after it reaches its peak value. The *ZT* value of the integrated Sb_2_Te_3_ thin film TE device was obtained as 0.12 ± 0.01. It is possible to make the device to go beyond 1.75 K of temperature difference. The material efficiency of the E-beam-grown Sb_2_Te_3_ thin film can be improved by thermal annealing and varying the film thickness for better phonon blocking and hole transmitting in the material, while the efficiency of the device can be further improved by improving the device fabrication such as decreasing the contact resistance.

## 4. Conclusions

The silicon diode temperature sensors and thermoelectric devices of Sb_2_Te_3_ thin film were fabricated successfully using microfabrication techniques. The electrical property of the fabricated silicon diode temperature sensor *versus* temperatures was characterized, showing that the diode temperature sensor is highly sensitive to the variation of temperatures. The Seebeck coefficient of Sb_2_Te_3_ and the figure merit of the integrated Sb_2_Te_3_ thin-film TE device were precisely measured from the devices. The fabrication of silicon diode temperature sensor and thermoelectric thin-film devices are compatible with the integrated circuit (IC) fabrication, and can be used for the applications of on-chip temperature monitoring, surface temperature stabilizing, and more.
